# Meteorological Factors Affecting Infectious Diarrhea in Different Climate Zones of China

**DOI:** 10.3390/ijerph191811511

**Published:** 2022-09-13

**Authors:** Mengya Yang, Can Chen, Xiaobao Zhang, Yuxia Du, Daixi Jiang, Danying Yan, Xiaoxiao Liu, Cheng Ding, Lei Lan, Hao Lei, Shigui Yang

**Affiliations:** 1 School of Public Health, School of Medicine, Zhejiang University, Hangzhou 310058, China; 2 State Key Laboratory for Diagnosis and Treatment of Infectious Diseases, Collaborative Innovation Center for Diagnosis and Treatment of Infectious Diseases, The First Affiliated Hospital College of Medicine, Zhejiang University, Hangzhou 310003, China

**Keywords:** infectious diarrhea, meteorological factors, fixed-effect model, Poisson regression, incidence

## Abstract

Meteorological factors and the increase in extreme weather events are closely related to the incidence rate of infectious diarrhea. However, few studies have explored whether the impact of the same meteorological factors on the incidence rate of infectious diarrhea in different climate regions has changed and quantified these changes. In this study, the time series fixed-effect Poisson regression model guided by climate was used to quantify the relationships between the incidence rate of various types of infectious diarrhea and meteorological factors in different climate regions of China from 2004 to 2018, with a lag of 0–2 months. In addition, six social factors, including per capita Gross Domestic Product (GDP), population density, number of doctors per 1000 people, proportion of urbanized population, proportion of children aged 0–14 years old, and proportion of elderly over 65 years old, were included in the model for confounding control. Additionally, the intercept of each province in each model was analyzed by a meta-analysis. Four climate regions were considered in this study: tropical monsoon areas, subtropical monsoon areas, temperate areas and alpine plateau areas. The results indicate that the influence of meteorological factors and extreme weather in different climate regions on diverse infectious diarrhea types is distinct. In general, temperature was positively correlated with all infectious diarrhea cases (0.2 ≤ r ≤ 0.6, *p* < 0.05). After extreme rainfall, the incidence rate of dysentery in alpine plateau area in one month would be reduced by 18.7% (95% confidence interval (CI): −27.8–−9.6%). Two months after the period of extreme sunshine duration happened, the incidence of dysentery in the alpine plateau area would increase by 21.9% (95% CI: 15.4–28.4%) in that month, and the incidence rate of typhoid and paratyphoid in the temperate region would increase by 17.2% (95% CI: 15.5–18.9%) in that month. The meta-analysis showed that there is no consistency between different provinces in the same climate region. Our study indicated that meteorological factors and extreme weather in different climate areas had different effects on various types of infectious diarrhea, particularly extreme rainfall and extreme sunshine duration, which will help the government develop disease-specific and location-specific interventions, especially after the occurrence of extreme weather.

## 1. Introduction

Infectious diarrhea is a preventable intestinal infectious disease, including cholera, dysentery, typhoid and paratyphoid and other infectious diarrheas (OIDs, defined as infectious diarrhea other than cholera, dysentery, typhoid or paratyphoid), but it is still a major public health problem in the world. According to the investigation of the World Health Organization, diarrheal disease causes about 1.8 million deaths every year [1], and the incidence of infectious diarrhea is more serious in developing countries [2,3]. China is a large developing country with a vast territory and complex and diverse climate. Even though the Chinese government provided tremendous subsidies for medical care and attached great importance to public health education, China is still facing a huge burden of infectious diarrhea. The incidence rate of diarrhea in China ranks among the top 15 in the world [4]. From January 2004 to December 2013, the annual incidence rate of infectious diarrhea accounted for 19.5% of the incidence rate of 45 recognized infectious diseases [5]. Various studies have shown that infectious diarrhea poses a great threat to public health in all age groups, especially infants and young children, and has caused a heavy economic burden to the Chinese government.

Previous studies have found that the incidence rate of infectious diarrhea is closely related to the seasonal changes [6,7,8,9,10], especially the meteorological conditions [11]. A large number of studies have shown that an increase in temperature or relative humidity will increase the incidence rate of infectious diarrhea [12,13,14,15,16]. Additionally, extreme weather events such as floods or extremely high temperatures have a great impact on infectious diarrhea in various places [17,18,19]. For example, studies in Taiwan found that the influence of extremely high temperature on the incidence rate of infectious diarrhea is mainly reflected in children and the elderly, while the incidence rate of infectious diarrhea caused by relative humidity and extreme rainfall is mainly reflected in adults [19]. Another study also showed that there is a significant positive correlation between the increase in the incidence rate of diarrhea and high temperature, high relative humidity and high accumulated rainfall 4 weeks before the date of visit [20].

Although the potential influencing meteorological factors of infectious diarrhea have been fully confirmed, no study has focused on whether the impact of the same meteorological factors on the incidence rate of infectious diarrhea in different climate regions has changed and quantified these changes, and whether these changes may lead to the high incidence rate of infectious diarrhea in some climate regions. The objective of this study was to estimate and quantify the casual associations between climate factors and the incidence of various types of infectious diarrhea in different climate regions by using the incidence rate of infectious diarrhea and climate variables in 31 provinces of China from 2004 to 2018, after adding some social factors related to the incidence rate of infectious diarrhea to control the confounding.

## 2. Materials and Methods

### 2.1. Surveillance Data

The monthly incidence rate (per 100,000 people) of infectious diarrhea (dysentery, typhoid and paratyphoid, cholera and other infectious diarrheas) in China and 31 provinces (excluding Hong Kong, Macao and Taiwan) from 2004 to 2018 was obtained from the recognized infectious disease report database opened by the public health and scientific data center of the Chinese Center for Disease Control (CDC) [21]. In 2003, the China Information System for Disease Control and Prevention (CISDCP), covering 31 provinces in the country, was established, and all data related to notifiable infectious diseases in China were uploaded to the CDC through the system [22].The data we obtained include the monthly number of new cases, incidence rate (per 100,000 people) and number of deaths and mortality (per 100,000 people) due to infectious diarrhea in various provinces of the country.

### 2.2. Meteorological Data

**Figure 1:**
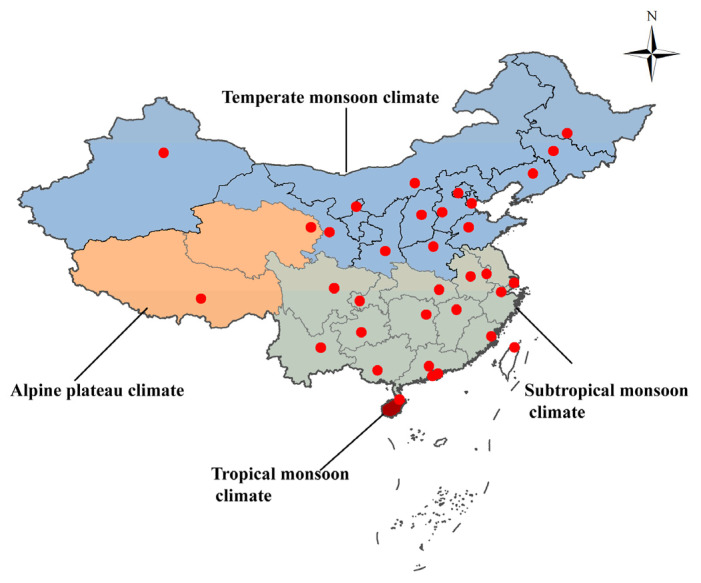
Distribution of climate types in China in this study. Red dots indicate the distribution of capital cities in the studied provinces.

Meteorological data of 31 provincial capitals were used to represent the climate of corresponding provinces (excluding Hong Kong, Macao and Taiwan). Using the monthly average meteorological data of the provincial capital cities to represent the meteorological situation of the whole province in a single month was relatively rough in space and time. However, in the absence of further detailed data, we can see from Figure 1 that the latitude of most provincial capitals is basically in the middle of the latitude range of the province, and the distribution of the climate zone is mainly determined by the latitude. The monthly average temperature (°C), relative humidity (RH) (%), rainfall (mm) and sunshine duration (hours) in 31 major cities in China from 2004 to 2008 were obtained from the China Statistical Yearbook [23]. Additionally, the monthly average temperature, RH, rainfall and sunshine duration in 31 major cities in China from 2009 to 2018 were obtained from the China Meteorological Administration [24]. The reason for having two different sources of meteorological data in this study was that the collection period for the data from the China Meteorological Administration, as the main meteorological data source in this study, began in 2009. Therefore, the meteorological data from 2004 to 2008 obtained from the China Statistical Yearbook were used as the supplementary data source. The database of the China Statistical Yearbook was derived from a data survey at the national level. Generally, the provincial and municipal statistical bureaus or survey teams are responsible for collecting the data and uploading it to the National Statistical Bureau. The data source was authentic and reliable.

There are five types of climates in China, including tropical monsoon climate, subtropical monsoon climate, temperate monsoon climate, temperate continental climate and alpine plateau climate. The tropical monsoon climate has no winter throughout the year, with high temperatures and plenty of rain. The annual accumulated temperature of the tropical monsoon climate is greater than 8000 °C. The average temperature in the coldest month is not less than 15 °C, and the average annual extreme minimum temperature is generally 0–5 °C. The subtropical monsoon climate has high temperatures and is rainy in summer, with mild temperatures and less rain in winter. The annual accumulated temperature of a subtropical monsoon climate is 4500–8000 °C. The average temperature in the coldest month is 0–15 °C. The temperate monsoon climate has high temperatures and rain in summer and is cold and dry in winter. The annual accumulated temperature of a temperate monsoon climate is 3000–4500 °C. The average temperature in the coldest month is −28–0 °C, and the average temperature in summer is mostly between 22 and 25 °C. The temperate continental climate, which includes most areas north of 40° north latitude in China, is dry and has less rain. The annual precipitation is 300–500 mm. The average temperature in summer is 26–27 °C, and the annual and daily temperature ranges are large. The alpine plateau climate is covered with snow and ice in winter and is cool and pleasant in summer. The annual accumulated temperature is less than 2000 °C, the daily average temperature is less than 10 °C, the maximum temperature is less than 5 °C, the daily temperature difference is large, but the annual temperature difference is small, the solar radiation is strong, and the sunshine is sufficient. The biggest difference between the temperate continental climate and temperate monsoon climate is the amount of summer rainfall. However, in the subsequent statistical analysis, we found that the incidence rate of infectious diarrhea caused by rainfall is not particularly significant, except for the occurrence of extreme rainfall. Therefore, the temperate monsoon climate and temperate continental climate of China were combined into the temperate climate, and then the total data were divided into four subsets according to the four climate types, which are tropical monsoon area, subtropical monsoon area, temperate area and alpine plateau area (Figure 1). There are two types of climates in some provinces at the same time. For example, the northern part of Jiangsu Province has a temperate climate, and the southern part has a tropical monsoon climate. For this situation, this paper divided it according to the climate type of the provincial capital city. In order to test the stability of the method of dividing the climate regions of the provinces according to the provincial capitals’ locations, the sensitivity analysis deleted Henan Province in the temperate climate region and Jiangsu Province, Anhui Province, and Sichuan Province in the subtropical region, and then performed the same modeling steps. The results are very similar (Supplementary Tables S12–S16), which indicates the robustness of the method.

The study also calculated the extremely high temperature, extreme rainfall and extreme sunshine duration from 2004 to 2018. In this study, the extreme climate conditions were defined as the month exceeding the 95th percentile of the data. The months with temperature, rainfall and sunshine duration exceeding the 95th percentile of the corresponding data were additionally marked as 1, and the months that did not exceed the 95th percentile of the corresponding data were marked as 0. In other words, extreme climate conditions were treated as the binary variable in this study.

### 2.3. Social Factor Data

The annual social factor data of 31 provinces (excluding Hong Kong, Macao and Taiwan) from 2004 to 2018 in the article were obtained from the China Statistical Yearbook [23]. The following six social factors affecting the incidence rate of infectious diarrhea were considered in this paper: per capita Gross Domestic Product (GDP) (RMB), number of doctors per 1000 people, population density (person/km^2^), proportion of urban population, proportion of children aged 0 to 14 in the total population and proportion of elderly over 65 years old in the total population.

### 2.4. Statistical Analysis

The Spearman correlation method was used to calculate the relationships between the incidence rate of infectious diarrhea and climate factors for the present or lag time with one or two months in different climate regions [19]. Meanwhile, the Spearman correlation method was also used to calculate the relationships between the monthly incidence rate of infectious diarrhea and social factors in different climate regions so as to preliminarily determine which factors in various climate regions were related to infectious diarrhea. Only meteorological factors with statistical significance and relatively strong correlation (r ≥ 0.15) were selected for inclusion in the model. In addition, each climate factor only selected the lag month when r was the largest and put it into the model. It is worth noting that when both the average and the extreme version of the same climate variable were both significantly correlated with the incidence rate of infectious diarrhea, the variable that produced the greatest R^2^ was included in the model. Because temperature and extremely high temperature, rainfall and extreme rainfall, sunshine duration and extreme sunshine duration are highly correlated, putting them into the model at the same time may lead to contradictory results. For the selection of social factors, because there were strong correlations between some social factors, we first selected the social factors which had strong correlations with the incidence rate of infectious diarrhea through Spearman correlation analysis, and at the same time, the correlation between social factors which entering the model was weak. In the Supplementary Materials, the parameters selected for infectious diarrhea models in various climate regions are shown in detail.

In this study, the time series fixed-effect Poisson regression model [19,25] was used to determine the best-fitting model related to infectious diarrhea and estimate the incidence rate of infectious diarrhea caused by climate factors. The regression model is described as follows:

(1) $$\begin{array}{ll} \ln\left(Y_{\mathrm{it}}\right)= & \alpha_{i0}+\alpha_{1}t+\alpha_{2}\sin\frac{2\pi t}{12}+\alpha_{3}T_{t-n}+\alpha_{4}{\mathrm{RH}}_{t-n}+\alpha_{5}{\mathrm{Rain}}_{t-n}\\ & +\alpha_{6}{\mathrm{Sunshine}}_{t-n}+\alpha_{7}T_{\mathrm{EXT},t-n}+\alpha_{8}{\mathrm{Rain}}_{\mathrm{EXT},t-n}\\ & +\alpha_{9}{\mathrm{Sunshine}}_{\mathrm{EXT},t-n}+\alpha_{10}{\mathrm{GDP}}_{t}+\alpha_{11}{\mathrm{HW}}_{t}+\alpha_{12}{\mathrm{PD}}_{t}\\ & +\alpha_{13}{\mathrm{PU}}_{t}+\alpha_{14}{\mathrm{PC}}_{t}+\alpha_{15}{\mathrm{PE}}_{t} \end{array}$$

where YY_i__t_ denotes the incidence rate of infectious diarrhea at time t (per 100,000 people) in province i, where α_1_ to α_15_, respectively, represent the coefficients. α_i0_ represents the intercept term of provinces in the climate region, i.e., the fixed effect in the model. That means the coefficient of various meteorological factors for infectious diarrhea is the same in the studied area of a certain climate type, and only the intercept term α_i0_ represents different provinces in the same climate-type area. T, RH, Rain, Sunshine, T_MAX_, Rain_EXT_ and Sunshine_EXT_ are monthly average temperature, relative humidity, rainfall, sunshine duration, extremely high temperature, extreme rainfall and extreme sunshine duration, respectively. Additionally, GDP, HW, PD, PU, PC and PE represent per capita GDP, number of doctors (per 1000), population density, proportion of urban population, proportion of children aged 0 to 14 in the total population and proportion of elderly over 65 years old in the total population, respectively. The term t−n in the subscript represents the n-month lag time, where n is 0, 1, 2. Considering the seasonality and long-term trends which may be associated with weather conditions, the proposed model included a triangular function, sin(2πt/12), to reveal the seasonal component. Each province in China was divided according to the four climate types mentioned before, and the models of every type of infectious diarrhea were established for each climate type. The variables in each model were selected according to the results of Spearman’s correlation analysis. The parameter selection of each infectious diarrhea model in every climate region can be viewed in the Supplementary Materials. Finally, a meta-analysis was performed on all intercept items of each model, i.e., α_i0_, to further explore whether the differences between individual effects of provinces in the same climate region were statistically significant, so as to know whether the formulation of relevant prevention strategies needs to be adapted to local conditions.

The regression coefficients of climate variables (α_3_–α_9_) were transformed using the equation [19]:

100(e^α^ − 1) (2)

The equation could reveal the percentage change of infectious diarrhea incidence rate caused by the unit change of climate factors, including temperature, relative humidity, rainfall, sunshine duration, extremely high temperature, extreme rainfall and extreme sunshine duration.

All statistical analyses were carried out in R 3.63 (The R Project for Statistical Computing, Guangzhou, China).

## 3. Results

### 3.1. Burden and Trends of Infectious Diarrhea and Features of Meteorological Factors

**Figure 2:**
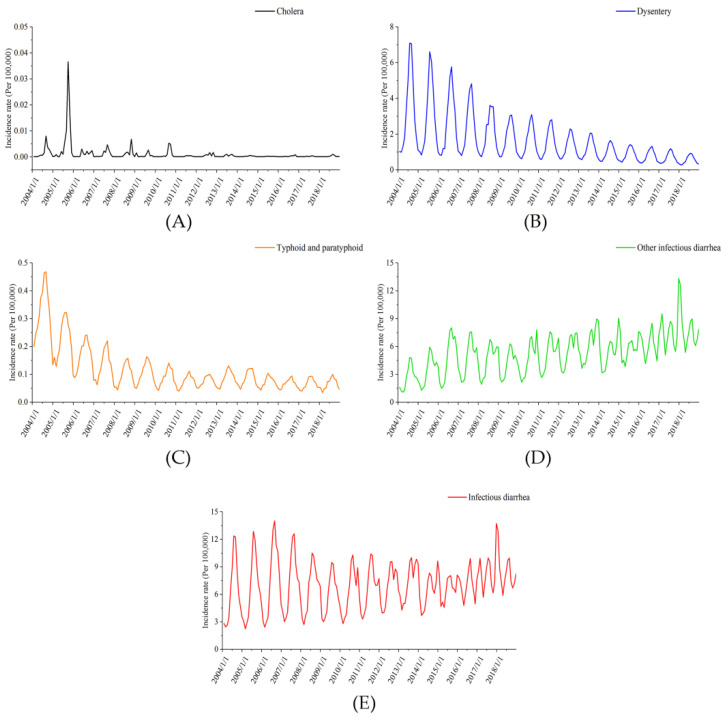
Incidence rate (per 100,000) of different types of infectious diarrhea in China from 2004 to 2018. (**A**) Cholera; (**B**) dysentery; (**C**) typhoid and paratyphoid; (**D**) other infectious diarrheas; (**E**) infectious diarrhea.

Figure 2 shows that from 2004 to 2018, the incidence rate of cholera was very low and there was no obvious periodicity, but the incidence rate of dysentery, typhoid and paratyphoid and other infectious diarrheas (OIDs) had an obvious cyclicity. Additionally, the incidence of dysentery and typhoid and paratyphoid represented a gradual downward trend, while the incidence rate of OIDs displayed a gradual upward trend. It can also be seen in Figure 1 that the incidence rate of all types of infectious diarrhea was high in summer and autumn.

Table 1 shows that from 2004 to 2018, the incidence rate of cholera was highest in the tropical monsoon region and the incidence rate of dysentery was higher in the temperate region and alpine plateau region. Meanwhile, the incidence rate of typhoid and paratyphoid was highest in the subtropical monsoon region, while the incidence rate of OIDs was relatively higher in the temperate region and subtropical monsoon region. Additionally, Table 2 shows the features of monthly climate factors from 2004 to 2018. The subtropical monsoon region and tropical monsoon region had relatively high monthly average temperatures, monthly RH and monthly rainfall. However, temperate and alpine plateau regions had a relatively high sunshine duration.

### 3.2. Correlation of Various Types of Infectious Diarrhea with Different Meteorological Factors

Spearman’s correlation analysis was conducted to quantify the relationships between the monthly incidence rate of infectious diarrhea and climate variables, with a lag of 0–2 months (Supplementary Tables S1–S5). The results indicate that in almost all climate regions, all types of infectious diarrhea had a significant positive correlation with monthly average temperature, except for OIDs in tropical monsoon regions. The influence of other meteorological factors on different types of infectious diarrhea varied greatly in different climate regions.

### 3.3. Effect of Different Meteorological Factors on Epidemic Features of the Various Types of Infectious Diarrhea

Supplementary Tables S7–S11 demonstrate the original coefficients for modeling the incidence rate of different types of infectious diarrhea in various climate regions. Table 3, Table 4, Table 5, Table 6 and Table 7 show the changes in the incidence rate of infectious diarrhea when the meteorological factors changed by one unit after the original coefficients of each model were converted by using Equation (2). In addition, the tables only show the coefficient conversion values of one meteorological factor that mainly affected the incidence rate of infectious diarrhea to avoid the Table 2 Fallacy [26]. It can be seen in Table 3 that only the model of the temperate region had an R^2^ greater than 0.5, while others were quite low. This may be because the incidence rate of cholera was extremely low, which leads to the unrepresentative results of these models.

The results in Table 4 show that the R^2^ of the regression models of all types of dysentery were quite high (greater than 0.7). The incidence rate of dysentery in all climate zones was positively correlated with the monthly average temperature. The incidence rate of dysentery in different climate areas would increase by 5.4–8.5% within that month when the monthly average temperature rose by one centigrade. In particular, the incidence rate of dysentery in alpine plateau areas was strongly influenced by extreme sunshine duration and extreme rainfall. Two months after extreme sunshine duration occurred, the incidence rate would rise by 21.9% (95% confidence interval (CI): 15.4–28.4%) within that month. After controlling the extreme sunshine duration, when extreme rainfall occurred, the incidence rate of dysentery would be reduced by 18.7% (95% CI: −27.8–−9.6%) in that month. The meta-analysis results of dysentery (Supplementary Figures S1–S4) in the temperate area show 0.3 (95% CI: −0.1–0.7). This means that even in the same temperate climate area, the dysentery prevention strategies in different provinces still need to be adjusted according to local conditions. Additionally, the meta-analysis results of dysentery in subtropical monsoon and alpine plateau regions show 1.8 (95% CI: 0.8–2.8) and 0.8 (95% CI: 0.3–1.3), which indicates the provinces in subtropical monsoon and alpine plateau regions had consistent individual effects.

Table 5 shows that R^2^ was relatively high in other climate regions except the tropical monsoon region and alpine plateau region. This may be because there are few data for the tropical monsoon area, and the incidence of typhoid and paratyphoid in alpine plateau areas is very low. The incidence rate of typhoid and paratyphoid increased by 3.0–8.0% within that month for every one centigrade rise in monthly average temperature in all climate regions. In the temperate region, two months after a period of extreme sunshine duration, the incidence of typhoid and paratyphoid increased by 17.2% (95% CI: 15.5–18.9%) within that month. The results of the meta-analysis (Supplementary Figures S1–S4) for typhoid and paratyphoid in the temperate region and subtropical monsoon area are −3.3 (95% CI: −3.9–−2.7) and −3.1 (95% CI: −3.6–−2.6), meaning that different intercept terms of provinces in temperate and subtropical monsoon zones were quite consistent in the individual effect, while the result of the meta-analysis in alpine plateau region was −12.0 (95% CI: −25.8–1.9), indicating that different provinces in alpine plateau region should take measures according to local conditions when formulating prevention and treatment strategies for typhoid and paratyphoid.

The results in Table 6 display that the R^2^ of the OIDs regression model in the tropical monsoon area was only 0.20, while the R^2^ in other climate areas was relatively high. This may be due to the small size of the data, since Hainan Province is the only tropical monsoon region in China. The incidence rate of OIDs increased by 3.9–4.6% within that month for every one centigrade rise in monthly average temperature in all climate regions. In the alpine plateau region, for every one hour increase in the sunshine duration, the incidence rate of OIDs increased by 0.5% (95% CI: 0.4–0.6) within that month. From the results of the meta-analysis (Supplementary Figures S1–S4), it could be seen that for OIDs, the results in the temperate region show 0.9 (95% CI: 0.0–1.8). This meant that when relevant departments in various provinces establish prevention strategies for OIDs, they should pay more attention to local conditions to better formulate prevention rules. Additionally, the results in subtropical monsoon and alpine plateau areas are −2.4 (95% CI: −2.9–−1.9) and −15.7 (95% CI: −26.0–−5.4), indicating that in these two climate regions, the individual effects were more consistent among those provinces.

Finally, the results in Table 7 show the modeling results after combining all types of infectious diarrhea. It could be seen that R^2^ in the tropical monsoon region was poor, and the other R^2^ was acceptable. As said before, the reason for this result was probably because there were too few data for the tropical monsoon region in China. For every one centigrade rise in monthly average temperature in all climate regions, the incidence rate of infectious diarrhea increased by 2.4–5.7% within that month. The results of the meta-analysis (Supplementary Figures S1–S4) in the temperate and subtropical monsoon areas are 1.4 (95% CI: 0.8–2.1), 1.3 (95% CI: 1.0–1.6). It could be seen that for infectious diarrhea, various cities in the same climate zone were quite uniform in the individual effect. Meanwhile, the result of the meta-analysis in the alpine plateau region is −0.3 (95% CI: −0.8–0.1), indicating that different provinces in the alpine plateau region should take measures according to local conditions when formulating prevention and treatment strategies for infectious diarrhea.

## 4. Discussion

This study found that climate change will have a great impact on human health [27]. Changes in temperature, humidity, rainfall and other climate factors will affect the incidence rate of influenza [28], diarrhea [29] and other infectious diseases [30], thus affecting public health and bringing financial burden to relevant government departments. Many studies have used the Poisson regression model to describe the incidence rate and hospitalization rate of diarrhea [29,31]. In this study, we also used fixed-effect time series Poisson regression to fit the relationships between meteorological factors and the incidence rate of various types of infectious diarrhea in different climate zones, and added some social factors to the model to control the confounding. The results show that the meteorological factors in different climate regions had different effects on the incidence rate of various infectious diarrheas, revealing that there was a certain lag relationship between the weather conditions and the incidence rate of infectious diarrhea. In general, the monthly average temperature had a positive correlation with all types of infectious diarrhea, which is consistent with previous studies [31,32,33]. At the same time, we found that extreme weather had a strong impact on the incidence rate of different infectious diarrheas in some climate regions. For example, extreme rainfall had a strong negative influence on the incidence rate of dysentery in the alpine plateau area, while extreme sunshine duration had an intense positive effect on the incidence rate of dysentery in the alpine plateau area and on the incidence rate of typhoid and paratyphoid in the temperate region.

This study revealed several important findings. First, the results quantify the impact of meteorological factors in different climate regions of China on different types of infectious diarrhea. The study found that temperature had a positive effect on all types of infectious diarrhea, which is not only consistent with previous studies [31,32,33], but also consistent with the pattern of high incidence rate of infectious diarrhea in summer and autumn. Second, the inclusion of extreme weather in the model allowed us to find that extreme rainfall and extreme sunshine duration had an intense impact on infectious diarrhea in temperate and alpine plateau areas, providing a scientific basis for policy makers to better prevent and control infectious diarrhea when facing extreme weather conditions. Third, the impact of climate change on the incidence rate of infectious diarrhea is indirectly caused in various other ways, so this study found that the impact of climate variables on the incidence rate of infectious diarrhea had a certain time lag. Zhang et al. [31] found that the impact of maximum and minimum temperature on the incidence rate of diarrhea had a 0-month lag time in Townsville with tropical climate, while the lag effects of rainfall on the incidence rate of diarrhea in Townsville was 3 months. Chou et al. [19] showed that the impact of the highest monthly temperature and monthly relative humidity on the incidence rate of diarrhea in Taiwan had a 1-month lag, while extreme rainfall had a 2-month lag. These indicate that local climate conditions affect the impact of meteorological factors on the incidence rate of infectious diarrhea, and strategies for preventing and controlling infectious diarrhea should be adapted to local conditions. The results of the meta-analysis also indicate that there is no consistency between individual effects of different provinces even in the same climate region. There are many reasons explaining this phenomenon, such as the differences in climate, social development, personal health level and so on among various provinces. These emphasize again that the prevention policy for infectious diarrhea should be adjusted according to the local special conditions.

The causal association between infectious diarrhea and meteorological conditions can be explained by pathogen activity. Infectious diarrhea is easily caused by microorganisms, which is closely related to environmental conditions [34,35]. Changes in rainfall and temperature are related to fecal contamination [36], which is the direct cause of infectious diarrhea [37]. Extreme rainfall and extreme sunshine duration may affect human behavior patterns and even lead to floods or droughts and other disasters, leading to population displacement and subsequent health problems [36]. Meanwhile, the causal association between meteorological factors and infectious diarrhea is affected by many confounding factors, such as personal hygiene, latrine utilization, water availability and quality, which are also very important factors affecting the incidence rate of infectious diarrhea. In this paper, the fixed-effect model was used to fit the relationship between the incidence rate of infectious diarrhea and the meteorological factors in various climatic regions, and the addition of some social factors in the model could control confounding to a certain extent. However, the model could not include all possible confounding factors, so the parameter estimation results in this paper are only approximate estimates of the changes in the incidence rate of infectious diarrhea caused by meteorological factors. The findings would be more convincing if the same results as those in this paper could be obtained in populations with different potential confounding patterns but the same or similar exposure patterns [38].

This study had several limitations. First, this study only obtained the monthly incidence rate of infectious diarrhea and the monthly data of meteorological factors rather than the daily data, and could not more accurately analyze the lag effect of climate factors on the incidence rate of infectious diarrhea. Additionally, the article used provincial data rather than county/district-level or the prefecture-level data, so we were unable to conduct a more detailed analysis, and the results lack certain reliability. More detailed data about meteorological factors and the incidence of infectious diarrhea are needed. Second, the method of using the monthly meteorological data of the provincial capital city to represent the monthly meteorological data of the whole province was not rigorous enough, but since we lacked more detailed meteorological data, there was no way to verify the rationality of this method with actual data. In the future, we will strive to obtain more detailed meteorological data to further confirm our findings. Third, compared with temperate and subtropical monsoon regions, there are few data for alpine plateau and tropical monsoon regions, which leads to a low R^2^, and the impact of meteorological factors on infectious diarrhea in these two climate regions cannot be studied thoroughly. According to the existing research, extremely high or low temperatures and rainfall will affect the incidence rate of infectious diarrhea in Townsville with tropical climate [31]. Additionally, extremely high temperatures and more sunshine before the monsoon will increase the first summit of infectious diarrhea in the tropical region [39]. Moreover, a study has shown that higher ambient temperatures will affect rainfall, thus increasing the risk of diarrhea in rural areas in southern India [40]. These provide a good direction and basis for our future research. However, there are few studies focusing on the relationship between the incidence rate of infectious diarrhea and meteorological factors in alpine plateau climate areas. More data on these two climate regions, especially on alpine plateau climate areas, are required for further research in the future. In addition, the incidence rate of cholera in China is very low and does not show obvious periodicity, which makes it difficult to study the environmental impact factors of cholera and achieve good representative results. Additionally, social factors are also important factors affecting the incidence rate of infectious diarrhea. The six social factors considered in this paper are far from enough. As this article involves multiple cities, we temporary could not obtain all relevant social factor data for this research area. We believe that this is also one of the main reasons why the meta-analysis showed that the individual effects of different provinces in the same climate region were statistically significant. In the future, more relevant social factors can be incorporated into the model to obtain more accurate results. Finally, in ecological research, it is difficult to control confounding factors, link individual outcome events with individual exposure or co-variate history and determine causality. Moreover, if the background risk distribution (in this study, this refers to other factors affecting the incidence rate of infectious diarrhea, such as the various social factors mentioned above) is not fully controlled, the ecological study may reach a spurious association [41]. However, it is very difficult to control the background risk distribution in the ecological research of multiple regions, because the interactions between the factors in different regions vary. In this study, Spearman correlation analysis was used to screen the meteorological factors in order to minimize the problem of multi-collinearity among variables, a fixed-effect model was used, and some social factors were included in the model to control some confounding factors to some extent. Additionally, we were very clear that these methods are not enough to achieve sufficiently reliable results. Thus, individual-level studies and more detailed research are needed in the future.

## 5. Conclusions

Our study demonstrated that meteorological factors in different climate regions in China have different effects on various types of infectious diarrhea. There was a uniform positive correlation between temperature and the incidence rate of infectious diarrhea. Extreme rainfall had a strong negative influence on the incidence rate of dysentery in alpine plateau areas. Extreme sunshine durations imposed an intense positive effect on the incidence rate of dysentery in alpine plateau areas and the incidence rate of typhoid and paratyphoid in temperate areas. The meteorological factors affecting various types of infectious diarrhea in different climate regions were identified in this study, which will help the government to formulate interventions for specific infectious diarrhea and specific locations.

## Figures and Tables

**Table 1:** Description of monthly incidence rate (per 100,000) of infectious diarrhea in China from 2004 to 2018.

	Mean ± SD	Minimum	Maximum	IQR
**Cholera** *				
China	0.1 ± 1.7	0.0	83.8	0.0
Temperate region	0.1 ± 0.5	0.0	14.9	0.0
Subtropical monsoon region	0.4 ± 14.0	0.0	700.0	0.0
Tropical monsoon region	0.9 ± 7.4	0.0	82.8	0.0
Alpine plateau region	0.0 ± 0.0	0.0	0.0	0.0
**Dysentery**				
China	2.2 ± 3.8	0.0	57.2	1.7
Temperate region	3.1 ± 5.2	0.1	57.2	2.3
Subtropical monsoon region	1.3 ± 1.4	0.0	11.2	1.3
Tropical monsoon region	0.8 ± 0.6	0.1	3.4	0.8
Alpine plateau region	3.0 ± 2.9	0.4	18.9	2.4
**Typhoid and paratyphoid**				
China	0.1 ± 0.3	0.0	3.5	0.1
Temperate region	0.0 ± 0.1	0.0	1.4	0.0
Subtropical monsoon region	0.2 ± 0.4	0.0	3.5	0.1
Tropical monsoon region	0.0 ± 0.0	0.0	0.1	0.0
Alpine plateau region	0.0 ± 0.1	0.0	0.9	0.0
**Other infectious diarrheas**				
China	5.7 ± 8.1	0.0	124.8	5.0
Temperate region	6.7 ± 10.4	0.0	124.8	6.2
Subtropical monsoon region	5.5 ± 5.7	0.0	45.7	4.6
Tropical monsoon region	3.3 ± 1.6	0.7	11.5	1.5
Alpine plateau region	2.2 ± 2.7	0.0	15.2	3.5
**Infectious diarrhea**				
China	8.1 ± 10.8	0.3	154.5	6.1
Temperate region	9.8 ± 14.7	0.3	154.5	8.1
Subtropical monsoon region	7.0 ± 5.9	0.4	50.1	5.4
Tropical monsoon region	4.2 ± 1.7	1.5	14.0	1.1
Alpine plateau region	5.2 ± 3.4	0.5	10.3	4.4

* Represents the incidence rate of cholera per 10,000,000 people.

**Table 2:** Description of monthly meteorological factors in China from 2004 to 2018.

	Mean ± SD	Minimum	Maximum	IQR
**Temperature (** **°C** **)**				
China	14 ± 11	-23.	33	16
Temperate region	11 ± 12	-23.	30	21
Subtropical monsoon region	18 ± 8	-2	33	13
Tropical monsoon region	25 ± 4	13	30	7
Alpine plateau region	6 ± 8	-11	20	14
**Relative humidity (%)**				
China	65 ± 14	14	94	21
Temperate region	57 ± 13	22	92	20
Subtropical monsoon region	74 ± 8	42	92	10
Tropical monsoon region	81 ± 4	71	94	5
Alpine plateau region	51 ± 15	14	77	22
**Rainfall (mm)**				
China	75 ± 92	0	1213	93
Temperate region	41 ± 55	0	422	52
Subtropical monsoon region	109 ± 100	0	835	114
Tropical monsoon region	157 ± 177	0	1213	203
Alpine plateau region	37 ± 45	0	230	60
**Sunshine duration (hours)**				
China	168 ± 68	0	378	97
Temperate region	197 ± 55	17	363	74
Subtropical monsoon region	130 ± 61	0	378	88
Tropical monsoon region	157 ± 65	10	312	106
Alpine plateau region	227 ± 38	115	330	55

**Table 3:** The percentage of change in incidence rate of cholera when meteorological factors changed by one unit.

	Incidence Rate Change (%)	R^2^
**China**		0.23
Rainfall (mm)	0.4 (±0.0)	
**Temperate region**		0.54
Temperature (lag 1) * (°C)	46.7 (±3.7)	
**Subtropical monsoon region**		0.18
Temperature (°C)	5.4 (±2.7)	
**Tropical monsoon region**		0.26
Relative humidity (lag 2) * (%)	−25.5(±15.6)	

* Lag 1/2 represent the lag effects of 1 or 2 months.

**Table 4:** The percentage of change in incidence rate of dysentery when meteorological factors changed by one unit.

	Incidence Rate Change (%)	R^2^
**China**		0.87
Temperature (°C)	5.6 (±0.1)	
**Temperate region**		0.90
Temperature (°C)	5.6 (±0.1)	
**Subtropical monsoon region**		0.77
Temperature (°C)	5.4 (±0.1)	
**Tropical monsoon region**		0.78
Temperature (lag 1) * (°C)	8.5 (±1.1)	
**Alpine plateau region**		0.76
Extreme rainfall	−18.7 (±9.1)	
Extreme sunshine duration (lag 2) *	21.9 (±6.5)	

* Lag 1/2 represent the lag effects of 1 or 2 months.

**Table 5:** The percentage of change in incidence rate of typhoid and paratyphoid when meteorological factors changed by one unit.

	Incidence Rate Change (%)	R^2^
**China**		0.83
Temperature (°C)	3.0 (±0.2)	
**Temperate region**		0.71
Extreme sunshine duration (lag 2) *	17.2 (±1.7)	
**Subtropical monsoon region**		0.82
Temperature (°C)	4.1 (±0.3)	
**Tropical monsoon region**		0.17
Temperature (°C)	5.8 (±1.6)	
**Alpine plateau region**		0.03
Temperature (°C)	8.0 (±3.1)	

* Lag 1/2 represent the lag effects of 1 or 2 months.

**Table 6:** The percentage of change in incidence rate of other infectious diarrheas when meteorological factors changed by one unit.

	Incidence Rate Change (%)	R^2^
**China**		0.73
Temperature (lag 1) * (°C)	3.9 (±0.1)	
**Temperate region**		0.79
Temperature (lag 1) * (°C)	4.9 (±0.2)	
**Subtropical monsoon region**		0.61
Temperature (lag 2) * (°C)	4.6 (±0.5)	
**Tropical monsoon region**		0.20
**Alpine plateau region**		0.74
Sunshine duration (hours)	0.5 (±0.1)	

* Lag 1/2 represented the lag effects of 1 or 2 months.

**Table 7:** When meteorological factors change by one unit, the percentage of change in incidence rate of infectious diarrhea.

	Incidence Rate Change (%)	R^2^
**China**		0.76
Temperature (lag 1) * (°C)	5.5 (±0.1)	
**Temperate region**		0.83
Temperature (lag 1) * (°C)	5.7 (±0.2)	
**Subtropical monsoon region**		0.57
Temperature (lag 1) * (°C)	2.4 (±0.3)	
**Tropical monsoon region**		0.10
**Alpine plateau region**		0.47
Temperature(lag 1) * (°C)	4.0 (±0.6)	

* Lag 1/2 represent the lag effects of 1 or 2 months.

## Data Availability

Publicly available datasets were analyzed in this study. This data can be found here: Data-center of China Public Health Science. Available online: https://www.phsciencedata.cn/Share/index.jsp (accessed on 20 May 2022). China Statistical Yearbook. Available online: http://www.stats.gov.cn/tjsj/ndsj (accessed on 20 May 2022). China Meteorological Administration. Available online: http://data.cma.cn (accessed on 20 May 2022).

## Supplementary Materials

The following supporting information can be downloaded at: https://www.mdpi.com/article/10.3390/ijerph191811511/s1. Supplementary Table S1. Correlations between the incidence rate of infectious diarrhea and climate variables in China from 2004 to 2018. Supplementary Table S2. Correlations between the incidence rate of infectious diarrhea and climate variables in temperate region from 2004 to 2018. Supplementary Table S3. Correlations between the incidence rate of infectious diarrhea and climate variables in subtropical monsoon region from 2004 to 2018. Supplementary Table S4. Correlations between the incidence rate of infectious diarrhea and climate variables in tropical monsoon region from 2004 to 2018. Supplementary Table S5. Correlations between the incidence rate of infectious diarrhea and climate variables in alpine plateau region from 2004 to 2018. Supplementary Table S6. Correlations between the incidence rate of infectious diarrhea and social variables in China and in different climate regions from 2004 to 2018. Supplementary Table S7. Parameters from Poisson regression model for the incidence rate of cholera in different climate zones from 2004 to 2018. Supplementary Table S8. Parameters from Poisson regression model for the incidence rate of dysentery in China and in different climate zones from 2004 to 2018. Supplementary Table S9. Parameters from Poisson regression model for the incidence rate of typhoid and paratyphoid in China and in different climate zones from 2004 to 2018. Supplementary Table S10. Parameters from Poisson regression model for the incidence rate of other infectious diarrhea in China and in different climate zones from 2004 to 2018. Supplementary Table S11. Parameters from Poisson regression model for the incidence rate of infectious diarrhea in China and in different climate zones from 2004 to 2018. Supplementary Table S12. Parameters from Poisson regression model for the incidence rate of cholera in different climate zones from 2004 to 2018. Supplementary Table S13. Parameters from Poisson regression model for the incidence rate of dysentery in China and in different climate zones from 2004 to 2018. Supplementary Table S14. Parameters from Poisson regression model for the incidence rate of typhoid and paratyphoid in China and in different climate zones from 2004 to 2018. Supplementary Table S15. Parameters from Poisson regression model for the incidence rate of other infectious diarrhea in China and in different climate zones from 2004 to 2018. Supplementary Table S16. Parameters from Poisson regression model for the incidence rate of infectious diarrhea in China and in different climate zones from 2004 to 2018. Supplementary Figure S1. Meta-analysis of different types of infectious diarrhea in China. (A) Cholera; (B) dysentery; (C) typhoid and paratyphoid; (D) other infectious diarrhea; (E) infectious diarrhea. Supplementary Figure S2. Meta-analysis of different types of infectious diarrhea in temperate region. (A) Cholera; (B) dysentery; (C) typhoid and paratyphoid; (D) other infectious diarrhea; (E) infectious diarrhea. Supplementary Figure S3. Meta-analysis of different types of infectious diarrhea in subtropical monsoon region. (A) Cholera; (B) dysentery; (C) typhoid and paratyphoid; (D) other infectious diarrhea; (E) infectious diarrhea. Supplementary Figure S4. Meta-analysis of different types of infectious diarrhea in alpine plateau region. (A) Dysentery; (B) typhoid and paratyphoid; (C) other infectious diarrhea; (D) infectious diarrhea.
