# Supplementary material for: Meteorological Factors Affecting Infectious Diarrhea in Different Climate Zones of China
Source: Int J Environ Res Public Health. 2022 Sep 13;19(18):11511. doi: 10.3390/ijerph191811511 (PMC9517640; doi:10.3390/ijerph191811511)
Supplement: Supplementary file 1 [file ijerph-19-11511-s001.zip › ijerph-1832140-supplementary.pdf]

Supplementary Materials

Supplementary Table S1. Correlations between the incidence rate of infectious diarrhea and climate variables in China from 2004 to 2018.

Climate variables	Lag (months)	Cholera		Dysentery		Typhoid and paratyphoid		Other infectious diarrheas		Infectious diarrhea	
		**r**	**p-value**	**r**	**p-value**	**r**	**p-value**	**r**	**p-value**	**r**	**p-value**
Average temperature	0	0.29	<0.05	0.30	<0.05	0.40	<0.05	0.34	<0.05	0.40	<0.05
	1	0.31	<0.05	0.29	<0.05	0.39	<0.05	0.39	<0.05	0.44	<0.05
	2	0.26	<0.05	0.17	<0.05	0.33	<0.05	0.37	<0.05	0.39	<0.05
Average relative humidity (%)	0	0.13	<0.05	-0.14	<0.05	0.40	<0.05	0.12	<0.05	0.07	<0.05
	1	0.10	<0.05	-0.21	<0.05	0.37	<0.05	0.10	<0.05	0.03	<0.05
	2	0.07	<0.05	-0.28	<0.05	0.35	<0.05	0.08	<0.05	-0.01	0.27
Rainfall (mm)	0	0.20	<0.05	0.07	<0.05	0.36	<0.05	0.13	<0.05	0.17	<0.05
	1	0.11	<0.05	0.07	<0.05	0.35	<0.05	0.16	<0.05	0.20	<0.05
	2	0.12	<0.05	-0.01	0.65	0.36	<0.05	0.18	<0.05	0.19	<0.05
Sunshine duration (hours)	0	-0.02	0.10	0.29	<0.05	-0.18	<0.05	-0.03	0.05	0.07	<0.05
	1	0.02	0.23	0.33	<0.05	-0.18	<0.05	0.02	0.26	0.13	<0.05
	2	0.02	0.11	0.32	<0.05	-0.20	<0.05	0.02	0.11	0.13	<0.05
Max temperature	0	0.16	<0.05	0.03	<0.05	0.17	<0.05	0.11	<0.05	0.11	<0.05
	1	0.23	<0.05	0.02	<0.05	0.16	<0.05	0.12	<0.05	0.11	<0.05
	2	0.19	<0.05	0.01	0.61	0.13	<0.05	0.11	<0.05	0.09	<0.05
Extreme rainfall (mm)	0	0.11	<0.05	-0.05	<0.05	0.16	<0.05	0.07	<0.05	0.05	<0.05
	1	0.11	<0.05	-0.05	<0.05	0.16	<0.05	0.08	<0.05	0.06	<0.05

	2	0.12	<0.05	-0.07	<0.05	0.15	<0.05	0.09	<0.05	0.06	<0.05
Extreme sunshine duration (hours)	0	-0.02	0.09	0.17	<0.05	0.04	<0.05	0.04	<0.05	0.09	<0.05
	1	0.01	0.40	0.19	<0.05	0.05	<0.05	0.06	<0.05	0.12	<0.05
	2	0.01	0.51	0.19	<0.05	0.05	<0.05	0.06	<0.05	0.12	<0.05

Supplementary Table S2. Correlations between the incidence rate of infectious diarrhea and climate variables in temperate region from 2004 to 2018.

**Climate variables**	**Lag (months)**	**Cholera**		**Dysentery**		**Typhoid and paratyphoid**		**Other infectious diarrheas**		**Infectious diarrhea**	
		**r**	**p-value**	**r**	**p-value**	**r**	**p-value**	**r**	**p-value**	**r**	**p-value**
Average temperature	0	0.24	<0.05	0.59	<0.05	0.26	<0.05	0.39	<0.05	0.50	<0.05
	1	0.25	<0.05	0.59	<0.05	0.25	<0.05	0.42	<0.05	0.54	<0.05
	2	0.20	<0.05	0.43	<0.05	0.18	<0.05	0.38	<0.05	0.45	<0.05
Average relative humidity (%)	0	0.11	<0.05	0.10	<0.05	0.06	<0.05	-0.01	0.54	0.03	0.20
	1	0.06	<0.05	-0.05	<0.05	-0.02	0.29	-0.06	<0.05	-0.07	<0.05
	2	-0.04	<0.05	-0.20	<0.05	-0.11	<0.05	-0.12	<0.05	-0.17	<0.05
Rainfall (mm)	0	0.18	<0.05	0.35	<0.05	0.19	<0.05	0.09	<0.05	0.20	<0.05
	1	0.20	<0.05	0.34	<0.05	0.17	<0.05	0.13	<0.05	0.22	<0.05
	2	0.14	<0.05	0.20	<0.05	0.11	<0.05	0.11	<0.05	0.17	<0.05
Sunshine duration (hours)	0	0.00	0.99	0.23	<0.05	0.21	<0.05	0.10	<0.05	0.15	<0.05
	1	0.04	0.08	0.32	<0.05	0.26	<0.05	0.16	<0.05	0.24	<0.05
	2	0.10	<0.05	0.34	<0.05	0.25	<0.05	0.19	<0.05	0.27	<0.05
Max temperature	0	0.21	<0.05	0.20	<0.05	0.06	<0.05	0.23	<0.05	0.24	<0.05
	1	0.24	<0.05	0.20	<0.05	0.06	<0.05	0.22	<0.05	0.23	<0.05
	2	0.10	<0.05	0.15	<0.05	0.02	0.37	0.18	<0.05	0.18	<0.05
Extreme rainfall (mm)	0	0.17	<0.05	0.14	<0.05	0.04	<0.05	0.07	<0.05	0.11	<0.05
	1	0.13	<0.05	0.12	<0.05	0.04	0.06	0.04	<0.05	0.08	<0.05
	2	0.08	<0.05	0.05	<0.05	0.01	0.74	0.00	0.99	0.02	0.34
Extreme sunshine duration (hours)	0	0.03	0.12	0.15	<0.05	0.19	<0.05	0.13	<0.05	0.14	<0.05
	1	0.04	<0.05	0.19	<0.05	0.22	<0.05	0.16	<0.05	0.18	<0.05
	2	0.04	<0.05	0.17	<0.05	0.23	<0.05	0.15	<0.05	0.17	<0.05

Supplementary Table S3. Correlations between the incidence rate of infectious diarrhea and climate variables in subtropical monsoon region from 2004 to 2018.

**Climate variables**	**Lag (months)**	**Cholera**		**Dysentery**		**Typhoid and paratyphoid**		**Other infectious diarrheas**		**Infectious diarrhea**	
		**r**	**p-value**	**r**	**p-value**	**r**	**p-value**	**r**	**p-value**	**r**	**p-value**
Average temperature	0	0.30	<0.05	0.34	<0.05	0.29	<0.05	0.26	<0.05	0.33	<0.05
	1	0.33	<0.05	0.32	<0.05	0.26	<0.05	0.33	<0.05	0.40	<0.05
	2	0.27	<0.05	0.20	<0.05	0.17	<0.05	0.34	<0.05	0.38	<0.05
Average relative humidity (%)	0	0.00	0.87	0.04	0.07	0.06	<0.05	0.11	<0.05	0.10	<0.05
	1	-0.05	<0.05	0.01	0.76	0.06	<0.05	0.11	<0.05	0.09	<0.05
	2	-0.07	<0.05	-0.03	0.14	0.06	<0.05	0.13	<0.05	0.10	<0.05
Rainfall (mm)	0	0.14	<0.05	0.09	<0.05	0.14	<0.05	0.09	<0.05	0.12	<0.05
	1	0.19	<0.05	0.11	<0.05	0.15	<0.05	0.14	<0.05	0.17	<0.05
	2	0.19	<0.05	0.08	<0.05	0.14	<0.05	0.21	<0.05	0.22	<0.05
Sunshine duration (hours)	0	0.15	<0.05	0.11	<0.05	0.20	<0.05	0.04	<0.05	0.08	<0.05
	1	0.20	<0.05	0.12	<0.05	0.18	<0.05	0.08	<0.05	0.13	<0.05
	2	0.17	<0.05	0.07	<0.05	0.11	<0.05	0.08	<0.05	0.12	<0.05
Max temperature	0	0.13	<0.05	0.11	<0.05	0.05	<0.05	0.13	<0.05	0.13	<0.05
	1	0.21	<0.05	0.12	<0.05	0.04	0.07	0.13	<0.05	0.14	<0.05
	2	0.15	<0.05	0.09	<0.05	0.00	0.94	0.11	<0.05	0.12	<0.05
Extreme rainfall (mm)	0	0.08	<0.05	0.00	0.96	0.08	<0.05	0.08	<0.05	0.06	<0.05
	1	0.07	<0.05	0.01	0.62	0.10	<0.05	0.09	<0.05	0.08	<0.05
	2	0.08	<0.05	0.00	0.84	0.08	<0.05	0.11	<0.05	0.10	<0.05
Extreme sunshine duration (hours)	0	0.02	0.34	0.06	<0.05	0.14	<0.05	0.00	0.80	0.02	0.30
	1	0.09	<0.05	0.08	<0.05	0.13	<0.05	0.01	0.66	0.04	<0.05
	2	0.06	<0.05	0.08	<0.05	0.11	<0.05	-0.01	0.61	0.03	0.09

Supplementary Table S4. Correlations between the incidence rate of infectious diarrhea and climate variables in tropical monsoon region from 2004 to 2018.

**Climate variables**	**Lag (months)**	**Cholera**		**Dysentery**		**Typhoid and paratyphoid**		**Other infectious diarrheas**		**Infectious diarrhea**	
		**r**	**p-value**	**r**	**p-value**	**r**	**p-value**	**r**	**p-value**	**r**	**p-value**
Average temperature	0	0.08	0.28	0.23	<0.05	0.35	<0.05	-0.05	0.46	0.08	0.29
	1	0.13	0.07	0.25	<0.05	0.33	<0.05	0.05	0.53	0.21	<0.05
	2	0.16	<0.05	0.19	<0.05	0.19	<0.05	0.13	0.07	0.27	<0.05
Average relative humidity (%)	0	-0.12	0.12	-0.36	<0.05	-0.16	<0.05	0.10	0.16	-0.05	0.46
	1	-0.10	0.18	-0.29	<0.05	-0.06	0.41	0.04	0.61	-0.13	0.07
	2	-0.16	<0.05	-0.27	<0.05	0.05	0.52	0.04	0.56	-0.10	0.18
Rainfall (mm)	0	0.15	<0.05	0.12	0.11	0.21	<0.05	0.12	0.10	0.22	<0.05
	1	0.23	<0.05	0.10	0.17	0.26	<0.05	0.17	0.03	0.23	<0.05
	2	0.13	0.07	0.02	0.76	0.22	<0.05	0.28	<0.05	0.32	0.05
Sunshine duration (hours)	0	-0.02	0.76	0.09	0.22	0.35	<0.05	0.04	0.55	0.14	<0.05
	1	0.05	0.53	0.12	0.12	0.25	<0.05	0.13	0.08	0.23	<0.05
	2	0.13	0.07	0.07	0.34	0.20	<0.05	0.18	<0.05	0.23	<0.05
Max temperature	0	0.08	0.32	0.07	0.37	0.11	0.13	-0.12	0.12	-0.02	0.81
	1	0.07	0.32	0.08	0.31	0.08	0.26	-0.03	0.67	0.16	<0.05
	2	0.20	<0.05	0.08	0.61	-0.02	0.75	0.03	0.67	0.16	<0.05
Extreme rainfall (mm)	0	0.09	0.23	0.00	0.99	0.06	0.41	0.18	<0.05	0.16	<0.05
	1	0.09	0.25	-0.04	0.61	0.07	0.34	0.10	0.19	0.06	0.43
	2	-0.05	0.54	-0.05	0.54	0.10	0.19	0.08	0.28	0.09	0.25
Extreme sunshine duration (hours)	0	-0.05	0.54	-0.09	0.23	0.19	<0.05	0.02	0.77	-0.04	0.63
	1	-0.05	0.54	-0.09	0.24	0.06	0.42	0.09	0.21	0.05	0.47
	2	-0.05	0.54	-0.10	0.19	-0.02	0.78	0.07	0.38	0.03	0.72

Supplementary Table S5. Correlations between the incidence rate of infectious diarrhea and climate variables in alpine plateau region from 2004 to 2018.

Climate variables	Lag (months)	Dysentery		Typhoid and paratyphoid		Other infectious diarrheas		Infectious diarrhea	
		r	p-value	r	p-value	r	p-value	r	p-value
Average temperature	0	0.57	<0.05	0.18	<0.05	0.23	<0.05	0.59	<0.05
	1	0.60	<0.05	0.15	<0.05	0.29	<0.05	0.68	<0.05
	2	0.47	<0.05	0.08	0.14	0.30	<0.05	0.61	<0.05
Average relative humidity (%)	0	0.19	<0.05	0.18	<0.05	0.46	<0.05	0.51	<0.05
	1	0.10	0.06	0.14	<0.05	0.46	<0.05	0.44	<0.05
	2	-0.07	0.18	0.08	0.13	0.42	<0.05	0.29	<0.05
Rainfall (mm)	0	0.45	<0.05	0.12	<0.05	0.19	<0.05	0.46	<0.05
	1	0.47	<0.05	0.11	<0.05	0.24	<0.05	0.52	<0.05
	2	0.37	<0.05	0.02	0.68	0.24	<0.05	0.45	<0.05
Sunshine duration (hours)	0	0.26	<0.05	-0.17	<0.05	-0.38	<0.05	-0.09	0.07
	1	0.26	<0.05	-0.17	<0.05	-0.38	<0.05	-0.09	0.08
	2	0.27	<0.05	-0.15	<0.05	-0.37	<0.05	-0.06	0.28
Max temperature	0	0.19	<0.05	0.07	0.18	0.20	<0.05	0.28	<0.05
	1	0.21	<0.05	0.14	<0.05	0.21	<0.05	0.31	<0.05
	2	0.11	<0.05	0.16	<0.05	0.19	<0.05	0.24	<0.05
Extreme rainfall (mm)	0	0.21	<0.05	-0.07	0.21	-0.02	0.64	0.14	<0.05
	1	0.21	<0.05	0.01	0.88	-0.04	0.42	0.13	<0.05
	2	0.15	<0.05	-0.03	0.58	-0.05	0.30	0.06	0.25
Extreme sunshine duration (hours)	0	0.19	<0.05	-0.10	0.07	-0.13	<0.05	0.01	0.84
	1	0.20	<0.05	-0.16	<0.05	-0.16	<0.05	0.04	0.40
	2	0.21	<0.05	-0.07	0.18	-0.13	<0.05	0.09	0.08

Supplementary Table S6. Correlations between the incidence rate of infectious diarrhea and social variables in China and in different climate regions from 2004 to 2018.

Social variables	Cholera		Dysentery		Typhoid and paratyphoid		Other infectious diarrheas		Infectious diarrhea	
	**r**	**p-value**	**r**	**p-value**	**r**	**p-value**	**r**	**p-value**	**r**	**p-value**
**China**										
Per capita GDP (RMB)	0.03	<0.05	-0.43	<0.05	-0.26	<0.05	0.38	<0.05	0.13	<0.05
Number of doctors (per 1000)	-0.01	0.41	-0.34	<0.05	-0.26	<0.05	0.34	<0.05	0.14	<0.05
Population density (person/km^2)	0.22	<0.05	-0.13	<0.05	0.05	<0.05	0.37	<0.05	0.23	<0.05
Proportion of urban population	0.11	<0.05	-0.35	<0.05	-0.20	<0.05	0.34	<0.05	0.12	<0.05
Proportion of children aged 0 to 14	-0.10	<0.05	0.18	<0.05	0.33	<0.05	-0.08	<0.05	0.07	<0.05
Proportion of elderly over 65 years old	0.11	<0.05	-0.21	<0.05	-0.06	<0.05	0.19	<0.05	0.03	<0.05
**Temperate region**										
Per capita GDP (RMB)	0.10	<0.05	-0.22	<0.05	-0.33	<0.05	0.39	<0.05	0.20	<0.05
Number of doctors (per 1000)	0.14	<0.05	-0.12	<0.05	-0.15	<0.05	0.42	<0.05	0.26	<0.05
Population density (person/km^2)	0.18	<0.05	0.15	<0.05	-0.17	<0.05	0.46	<0.05	0.38	<0.05
Proportion of urban population	0.15	<0.05	-0.10	<0.05	-0.31	<0.05	0.25	<0.05	0.13	<0.05
Proportion of children aged 0 to 14	-0.14	<0.05	0.04	<0.05	0.38	<0.05	0.00	0.96	0.06	<0.05
Proportion of elderly over 65 years old	0.07	<0.05	-0.22	<0.05	-0.29	<0.05	0.16	<0.05	0.04	<0.05
**Subtropical monsoon region**										
Per capita GDP (RMB)	-0.02	0.32	-0.62	<0.05	-0.50	<0.05	0.29	<0.05	0.04	0.06
Number of doctors (per 1000)	-0.02	0.30	-0.63	<0.05	-0.34	<0.05	0.28	<0.05	0.04	0.03
Population density (person/km^2)	0.19	<0.05	-0.29	<0.05	-0.46	<0.05	0.14	<0.05	0.02	0.26
Proportion of urban population	0.09	<0.05	-0.56	<0.05	-0.45	<0.05	0.32	<0.05	0.09	<0.05
Proportion of children aged 0 to 14	-0.13	<0.05	0.34	<0.05	0.54	<0.05	-0.09	<0.05	0.07	<0.05
Proportion of elderly over 65 years old	0.03	0.14	-0.04	0.06	-0.60	<0.05	0.03	0.17	-0.08	<0.05

**Tropical monsoon region**										
Per capita GDP (RMB)	-0.19	<0.05	-0.89	<0.05	0.08	0.28	0.66	<0.05	0.34	<0.05
Number of doctors (per 1000)	-0.20	<0.05	-0.90	<0.05	0.09	0.21	0.64	<0.05	0.31	<0.05
Population density (person/km^2)	-0.19	<0.05	-0.89	<0.05	0.08	0.28	0.66	<0.05	0.34	<0.05
Proportion of urban population	-0.19	<0.05	-0.89	<0.05	0.08	0.28	0.66	<0.05	0.33	<0.05
Proportion of children aged 0 to 14	0.21	<0.05	0.70	<0.05	0.04	0.58	-0.53	<0.05	-0.25	<0.05
Proportion of elderly over 65 years old	0.27	<0.05	0.29	<0.05	0.10	<0.05	-0.01	0.94	0.14	<0.05
**Alpine plateau region**										
Per capita GDP (RMB)			-0.66	<0.05	0.05	0.35	0.38	<0.05	-0.09	0.09
Number of doctors (per 1000)			-0.65	<0.05	0.23	<0.05	0.64	<0.05	0.09	0.07
Population density (person/km^2)			-0.63	<0.05	0.32	<0.05	0.79	<0.05	0.23	<0.05
Proportion of urban population			-0.63	<0.05	0.32	<0.05	0.79	<0.05	0.23	<0.05
Proportion of children aged 0 to 14			0.50	<0.05	-0.21	<0.05	-0.62	<0.05	-0.26	<0.05
Proportion of elderly over 65 years old			-0.31	<0.05	0.22	<0.05	0.69	<0.05	0.48	<0.05

Abbreviations:GDP, Gross Domestic Product.

Infectious diarrhea models in different climatic regions:

National cholera model:

$$\ln(Y_{it}) = \alpha_{i0} + \alpha_1 t + \alpha_2 \sin\frac{2\pi t}{12} + \alpha_3 Rain_t + \alpha_4 T_{EXT,t-1} + \alpha_5 PD_t$$

National dysentery model:

$$\ln(Y_{it}) = \alpha_{i0} + \alpha_1 t + \alpha_2 \sin\frac{2\pi t}{12} + \alpha_3 T_t + \alpha_4 RH_{t-2} + \alpha_5 Sunshine_{t-1} + \alpha_6 GDP_t + \alpha_7 PE_t$$

National typhoid and paratyphoid model:

$$\ln(Y_{it}) = \alpha_{i0} + \alpha_1 t + \alpha_2 \sin\frac{2\pi t}{12} + \alpha_3 T_t + \alpha_4 RH_t + \alpha_5 Rain_t + \alpha_6 Sunshine_t + \alpha_7 PC_t$$

National other infectious diarrheas model:

$$\ln(Y_{it}) = \alpha_{i0} + \alpha_1 t + \alpha_2 \sin\frac{2\pi t}{12} + \alpha_3 T_{t-1} + \alpha_4 Rain_{t-2} + \alpha_5 GDP_t + \alpha_6 PD_t$$

National infectious diarrhea model:

$$\ln(Y_{it}) = \alpha_{i0} + \alpha_1 t + \alpha_2 \sin\frac{2\pi t}{12} + \alpha_3 T_{t-1} + \alpha_4 Rain_{t-1} + \alpha_5 PD_t$$

Temperate region cholera model:

$$\ln(Y_{it}) = \alpha_{i0} + \alpha_1 t + \alpha_2 \sin\frac{2\pi t}{12} + \alpha_3 T_{t-1} + \alpha_4 Rain_{t-1} + \alpha_5 PD_t + \alpha_6 PU_t$$

Temperate region dysentery model:

$$\ln(Y_{it}) = \alpha_{i0} + \alpha_1 t + \alpha_2 \sin\frac{2\pi t}{12} + \alpha_3 T_t + \alpha_4 RH_{t-2} + \alpha_5 Rain_t + \alpha_6 Sunshine_{t-2} + \alpha_7 GDP_t + \alpha_8 PD_t$$

Temperate region typhoid and paratyphoid model:

$$\ln(Y_{it}) = \alpha_{i0} + \alpha_1 t + \alpha_2 \sin\frac{2\pi t}{12} + \alpha_3 T_t + \alpha_4 Rain_t + \alpha_6 Sunshine_{t-2} + \alpha_7 PD_t + \alpha_8 PC_t$$

Temperate region other infectious diarrheas model:

$$\ln(Y_{it}) = \alpha_{i0} + \alpha_1 t + \alpha_2 \sin\frac{2\pi t}{12} + \alpha_3 T_{t-1} + \alpha_4 Sunshine_{t-2} + \alpha_5 PD_t + \alpha_6 HW_t$$

Temperate region infectious diarrhea model:

$$\ln(Y_{it}) = \alpha_{i0} + \alpha_1 t + \alpha_2 \sin\frac{2\pi t}{12} + \alpha_3 T_{t-1} + \alpha_4 RH_{t-2} + \alpha_5 Rain_{t-1} + \alpha_6 Sunshine_{t-2} + \alpha_7 HW_t + \alpha_8 PD_t$$

Subtropical monsoon region cholera model:

$$\ln(Y_{it}) = \alpha_{i0} + \alpha_1 t + \alpha_2 \sin\frac{2\pi t}{12} + \alpha_3 T_t + \alpha_4 Rain_{t-1} + \alpha_5 Sunshine_{t-1} + \alpha_6 PD_t$$

Subtropical monsoon region dysentery model:

$$\ln(Y_{it}) = \alpha_{i0} + \alpha_1 t + \alpha_2 \sin\frac{2\pi t}{12} + \alpha_3 T_t + \alpha_4 HW_t + \alpha_5 PD_t$$

Subtropical monsoon region typhoid and paratyphoid model:

$$\ln(Y_{it}) = \alpha_{i0} + \alpha_1 t + \alpha_2 \sin\frac{2\pi t}{12} + \alpha_3 T_t + \alpha_4 Rain_{t-1} + \alpha_6 Sunshine_t + \alpha_7 PC_t$$

Subtropical monsoon region other infectious diarrheas model:

$$\ln(Y_{it}) = \alpha_{i0} + \alpha_1 t + \alpha_2 \sin\frac{2\pi t}{12} + \alpha_3 T_{t-2} + \alpha_4 Rain_{t-2} + \alpha_5 PU_t$$

Subtropical monsoon region infectious diarrhea model:

$$\ln(Y_{it}) = \alpha_{i0} + \alpha_1 t + \alpha_2 \sin\frac{2\pi t}{12} + \alpha_3 T_{t-1} + \alpha_4 RH_{t-2} + \alpha_5 Rain_{t-1} + \alpha_6 Sunshine_{t-2}$$

Tropical monsoon region cholera model:

$$\ln(Y_t) = \alpha_0 + \alpha_1 t + \alpha_2 \sin\frac{2\pi t}{12} + \alpha_3 T_{t-2} + \alpha_4 RH_{t-2} + \alpha_5 Rain_{t-1} + \alpha_6 PC_t + \alpha_7 PE_t$$

Tropical monsoon region dysentery model:

$$\ln(Y_t) = \alpha_0 + \alpha_1 t + \alpha_2 \sin\frac{2\pi t}{12} + \alpha_3 T_{t-1} + \alpha_4 RH_t + \alpha_5 HW_t + \alpha_6 PE_t$$

Tropical monsoon region typhoid and paratyphoid model:

$$\ln(Y_t) = \alpha_0 + \alpha_1 t + \alpha_2 \sin\frac{2\pi t}{12} + \alpha_3 T_t + \alpha_4 RH_{t-1} + \alpha_5 Rain_{t-1} + \alpha_6 Sunshine_{EXT,t}$$

Tropical monsoon region other infectious diarrheas model:

$$\ln(Y_t) = \alpha_0 + \alpha_1 t + \alpha_2 \sin\frac{2\pi t}{12} + \alpha_3 Rain_{EXT,t} + \alpha_4 Sunshine_{t-2} + \alpha_5 PU_t$$

Tropical monsoon region infectious diarrhea model:

$$\ln(Y_t) = \alpha_0 + \alpha_1 t + \alpha_2 \sin\frac{2\pi t}{12} + \alpha_3 T_{EXT,t-1} + \alpha_4 Sunshine_{t-1} + \alpha_5 Rain_{EXT} + \alpha_6 PD_t$$

Alpine plateau region dysentery model:

$$\ln(Y_{it}) = \alpha_{i0} + \alpha_1 t + \alpha_2 \sin\frac{2\pi t}{12} + \alpha_3 T_{t-1} + \alpha_4 RH_t + \alpha_5 Rain_{EXT,t} + \alpha_6 Sunshine_{EXT,t-2} + \alpha_7 GDP_t + \alpha_8 PE_t$$

Alpine plateau region typhoid and paratyphoid model:

$$\ln(Y_{it}) = \alpha_{i0} + \alpha_1 t + \alpha_2 \sin\frac{2\pi t}{12} + \alpha_3 T_t + \alpha_4 RH_t + \alpha_5 Sunshine_t + \alpha_6 PD_t$$

Alpine plateau region other infectious diarrheas model:

$$\ln(Y_{it}) = \alpha_{i0} + \alpha_1 t + \alpha_2 \sin\frac{2\pi t}{12} + \alpha_3 T_{EXT,t-1} + \alpha_4 RH_t + \alpha_5 Rain_{t-1} + \alpha_6 Sunshine_t + \alpha_7 PD_t$$

Alpine plateau region infectious diarrhea model:

$$\ln(Y_{it}) = \alpha_{i0} + \alpha_1 t + \alpha_2 \sin\frac{2\pi t}{12} + \alpha_3 T_{t-1} + \alpha_4 RH_t + \alpha_5 Rain_{t-1} + \alpha_6 PE_t$$

Supplementary Table S7. Parameters from Poisson regression model for the incidence rate of cholera in China and in different climate zones from 2004 to 2018.

	Coefficient	Std. err.	p-value	R^2
**China**				0.23
Rainfall	0.0040	0.0003	<0.001	
Population density	0.0029	0.0012	0.016	
sin(2πt/12)	-2.2071	0.2168	<0.001	
Month	-0.0319	0.0029	<0.001	
**Temperate region**				0.55
Temperature (lag 1)*	0.3834	0.0359	<0.001	
Rainfall (lag 1)*	0.0051	0.0009	<0.001	
sin(2πt/12)	1.2870	0.2814	<0.001	
Month	-0.0280	0.0043	<0.001	
**Subtropical monsoon region**				0.18
Temperature	0.0527	0.0267	0.049	
sin(2πt/12)*	-2.0081	0.3272	<0.001	
Month	-0.0274	0.0039	<0.001	
**Tropical monsoon region**				0.40
Relative humidity (lag 2)*	-0.2948	0.1449	0.043	
Proportion of children aged 0-14	954.6000	459.3000	0.039	
Proportion of elderly over 65 years old	2612.0000	1178.0000	0.028	
sin(2πt/12)	-4.2190	1.3080	0.001	
Intercept	-426.0000	206.2000	0.040	

*Lag 1/2 represented the lag effects of 1 or 2 months.

Supplementary Table S8. Parameters from Poisson regression model for the incidence rate of dysentery in China and in different climate zones from 2004 to 2018.

	Coefficient	Std. err.	p-value	R^2
**China**				0.87
Temperature	0.0543	0.0008	<0.001	
Relative humidity (lag 2)*	-0.0020	0.0006	<0.001	
Proportion of elderly over 65 years old	3.4460	0.6185		
sin(2πt/12)	-0.3265	0.0099	<0.001	
Month	-0.0106	0.0003	<0.001	
**Temperate region**				0.90
Temperature	0.0545	0.0011	<0.001	
Relative humidity (lag 2)*	0.0029	0.0010	0.0069	
Rainfall	0.0003	0.0001	0.0166	
Sunshine duration (lag 2)*	0.0016	0.0003	<0.001	
sin(2πt/12)	-0.3416	0.0174	<0.001	

Month	-0.0119	0.0004	<0.001	
**Subtropical monsoon region**				0.77
Temperature	0.0526	0.0013	<0.001	
Number of doctors (Per 1,000)	-0.1294	0.0172	<0.001	
Population density	-0.0032	0.0002	<0.001	
sin(2πt/12)	-0.1590	0.0127	<0.001	
Month	-0.0070	0.0004	<0.001	
**Tropical monsoon region**				0.78
Temperature (lag 1)*	0.0817	0.0112	<0.001	
Relative humidity	0.0153	0.0070	0.029	
Number of doctors (Per 1,000)	-0.6105	0.1090	<0.001	
**Alpine plateau region**				0.76
Temperature (lag 1)*	0.0573	0.0060	<0.001	
Relative humidity	0.0156	0.0029	<0.001	
Extreme rainfall	-0.2073	0.0875	0.018	
Extreme sunshine duration (lag 2)*	0.1979	0.0626	0.002	
sin(2πt/12)	0.1717	0.5729	0.003	
Month	-0.0076	0.0024	0.002	

*Lag 1/2 represented the lag effects of 1 or 2 months.

Supplementary Table S9. Parameters from Poisson regression model for the incidence rate of typhoid and paratyphoid in China and in different climate zones from 2004 to 2018.

	**Coefficient**	**Std. err.**	**p-value**	**R^2**
**China**				0.83
Temperature	0.0298	0.0019	<0.001	
Relative humidity	0.0087	0.0016	<0.001	
Rainfall	0.0005	0.0001	<0.001	
Sunshine duration	0.0015	0.0003	<0.001	
Proportion of children aged 0-14	3.8787	0.7780	<0.001	
sin(2πt/12)	-0.1267	0.0166	<0.001	
Month	-0.0106	0.0002	<0.001	
**Temperate region**				0.71
Temperature	0.0249	0.0017	<0.001	
Extreme sunshine duration (lag 2)*	0.1590	0.0173	<0.001	
Proportion of children aged 0-14	-2.6810	1.2510	0.032	
sin(2πt/12)	-0.2216	0.0263	<0.001	
Month	-0.0086	0.0004	<0.001	
**Subtropical monsoon region**				0.82
Temperature	0.0399	0.0025	<0.001	
Rainfall (lag 1)*	0.0003	0.0001	0.011	
Proportion of children aged 0-14	4.7590	0.9418	<0.001	
sin(2πt/12)	-0.1053	0.0188	<0.001	

Month	-0.0096	0.0003	<0.001	
**Tropical monsoon region**				0.17
Temperature	0.0564	0.0163	<0.001	
Month	0.0026	0.0011	0.016	
Intercept	-3.9893	1.4262	0.005	
**Alpine plateau region**				0.03
Temperature	0.0772	0.0302	0.011	

*Lag 1/2 represented the lag effects of 1 or 2 months.

Supplementary Table S10. Parameters from Poisson regression model for the incidence rate of other infectious diarrheas in China and in different climate zones from 2004 to 2018.

	**Coefficient**	**Std. err.**	**p-value**	**R^2**
**China**				0.73
Temperature (lag 1)*	0.0387	0.0013	<0.001	
Rainfall (lag 2)*	-0.0010	0.0001	<0.001	
Population density	-0.0019	0.0001	<0.001	
Month	0.0085	0.0004	<0.001	
**Temperate region**				0.79
Temperature (lag 1)*	0.0476	0.0018	<0.001	
Sunshine duration (lag 2)*	0.0009	0.0002	<0.001	
Population density	-0.0024	0.0001	<0.001	
Number of doctors (Per 1,000)	0.0322	0.0095	<0.001	
sin(2πt/12)	0.1299	0.0236	<0.001	
Month	0.0035	0.0003	<0.001	
**Subtropical monsoon region**				0.61
Temperature (lag 2)*	0.0445	0.0049	<0.001	
Rainfall (lag 2)*	-0.0005	0.0001	<0.001	
Proportion of urbanized population	6.0715	0.6212	<0.001	
sin(2πt/12)	0.1926	0.0553	<0.001	
**Tropical monsoon region**				0.20
**Alpine plateau region**				0.74
Rainfall (lag 1)*	-0.0036	0.0016	0.020	
Sunshine duration	0.0045	0.0013	<0.001	
Population density	3.6299	0.9230	<0.001	
sin(2πt/12)	-0.6898	0.0948	<0.001	
Month	-0.0121	0.0048	0.013	

*Lag 1/2 represented the lag effects of 1 or 2 months.

Supplementary Table S11. Parameters from Poisson regression model for the incidence rate of infectious diarrhea in China and in different climate zones from 2004 to 2018.

	Coefficient	Std. err.	p-value	R^2
**China**				0.76
Temperature (lag 1)*	0.0535	0.0013	<0.001	
Rainfall (lag 1)*	-0.0005	0.0001	<0.001	
Population density	-0.0019	0.0001	<0.001	
sin(2πt/12)	0.1258	0.0160	<0.001	
Month	0.0007	0.0001	<0.001	
**Temperate region**				0.83
Temperature (lag 1)*	0.0556	0.0020	<0.001	
Rainfall (lag 1)*	0.0006	0.0002	<0.001	
Sunshine duration (lag 2)*	0.0010	0.0003	<0.001	
Population density	-0.0016	0.0001	<0.001	
Number of doctors (Per 1,000)	0.0305	0.0088	<0.001	
sin(2πt/12)	0.1684	0.0310	<0.001	
Month	-0.0012	0.0002	<0.001	
**Subtropical monsoon region**				0.57
Temperature (lag 1)*	0.0242	0.0027	<0.001	
Rainfall (lag 2)*	-0.0008	0.0001	<0.001	
sin(2πt/12)	-0.0799	0.0274	0.003	
Month	0.0018	0.0002	<0.001	
**Tropical monsoon region**				0.10
sin(2πt/12)	-0.2634	0.0745	<0.001	
**Alpine plateau region**				0.50
Temperature(lag 1)*	0.0392	0.0056	<0.001	
Proportion of elderly over 65 years old	26.7812	4.3054	<0.001	
Month	-0.0027	0.0005	<0.001	

*Lag 1/2 represented the lag effects of 1 or 2 months.

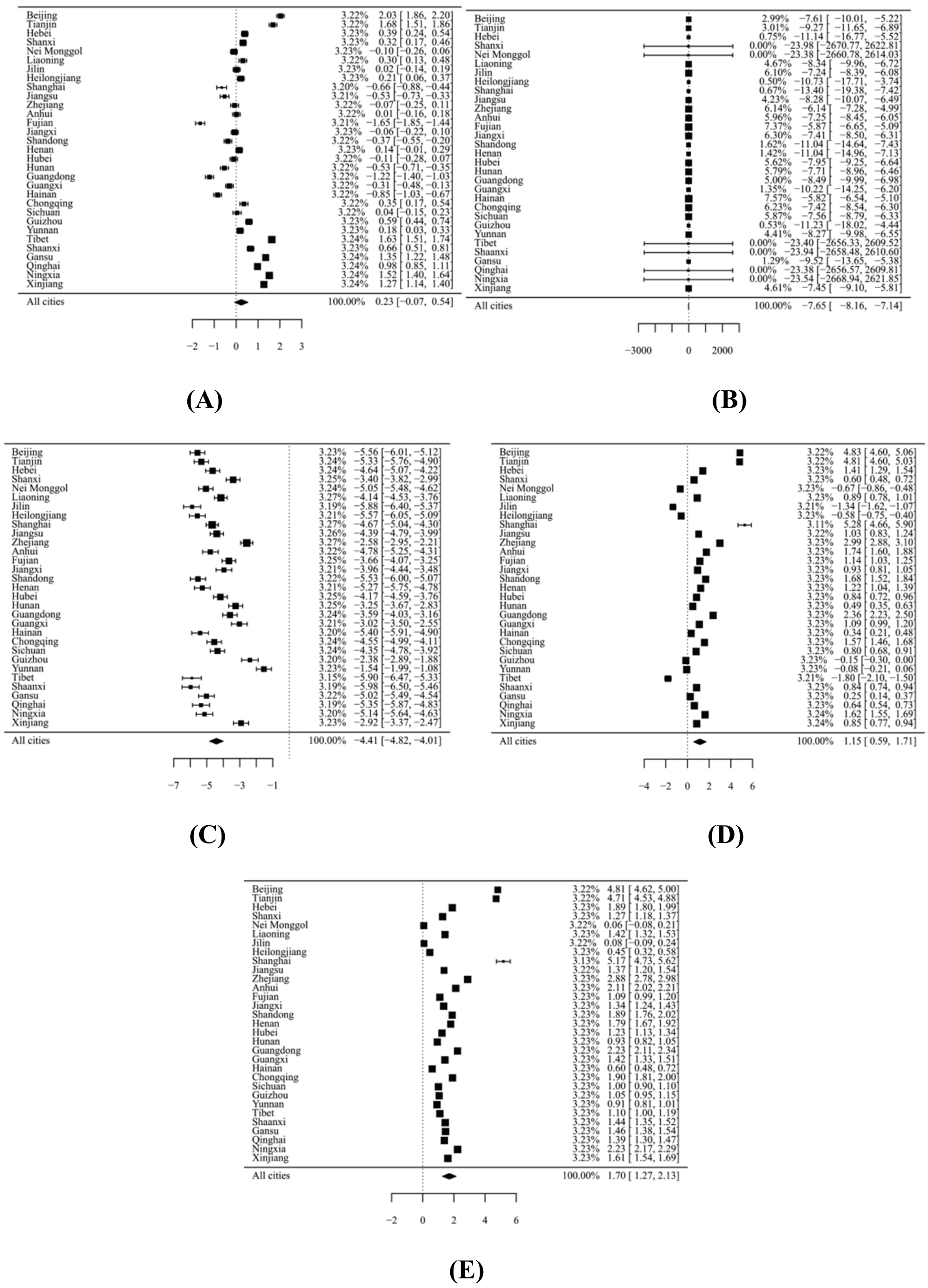

Supplementary Figure S1. Meta-analysis of different types of infectious diarrhea in China. (**A**) Cholera; (**B**) dysentery; (**C**) typhoid and paratyphoid; (**D**) other infectious diarrheas; (**E**) infectious diarrhea.

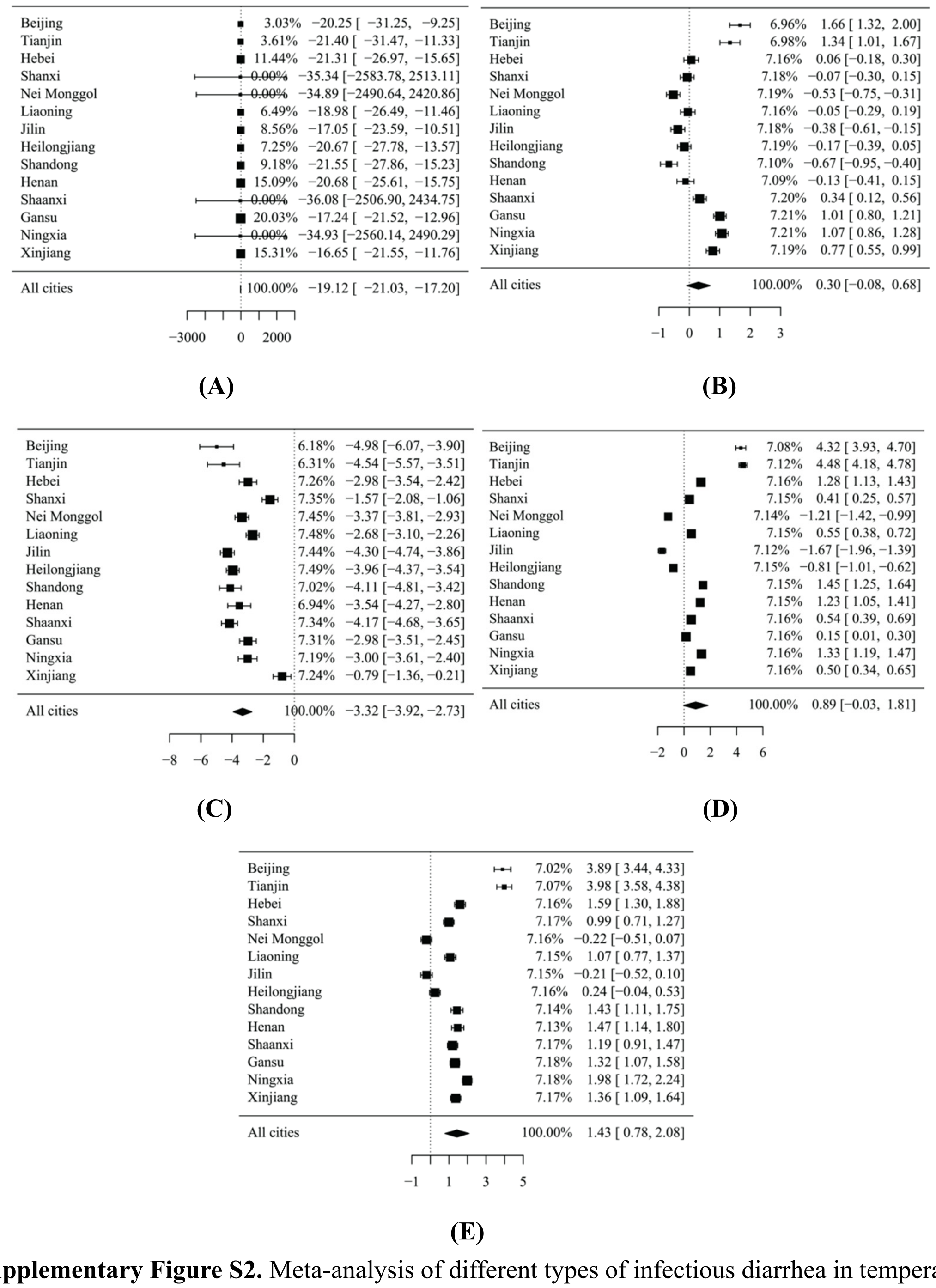

Supplementary Figure S2. Meta-analysis of different types of infectious diarrhea in temperate region. (**A**) Cholera; (**B**) dysentery; (**C**) typhoid and paratyphoid; (**D**) other infectious diarrheas; (**E**) infectious diarrhea.

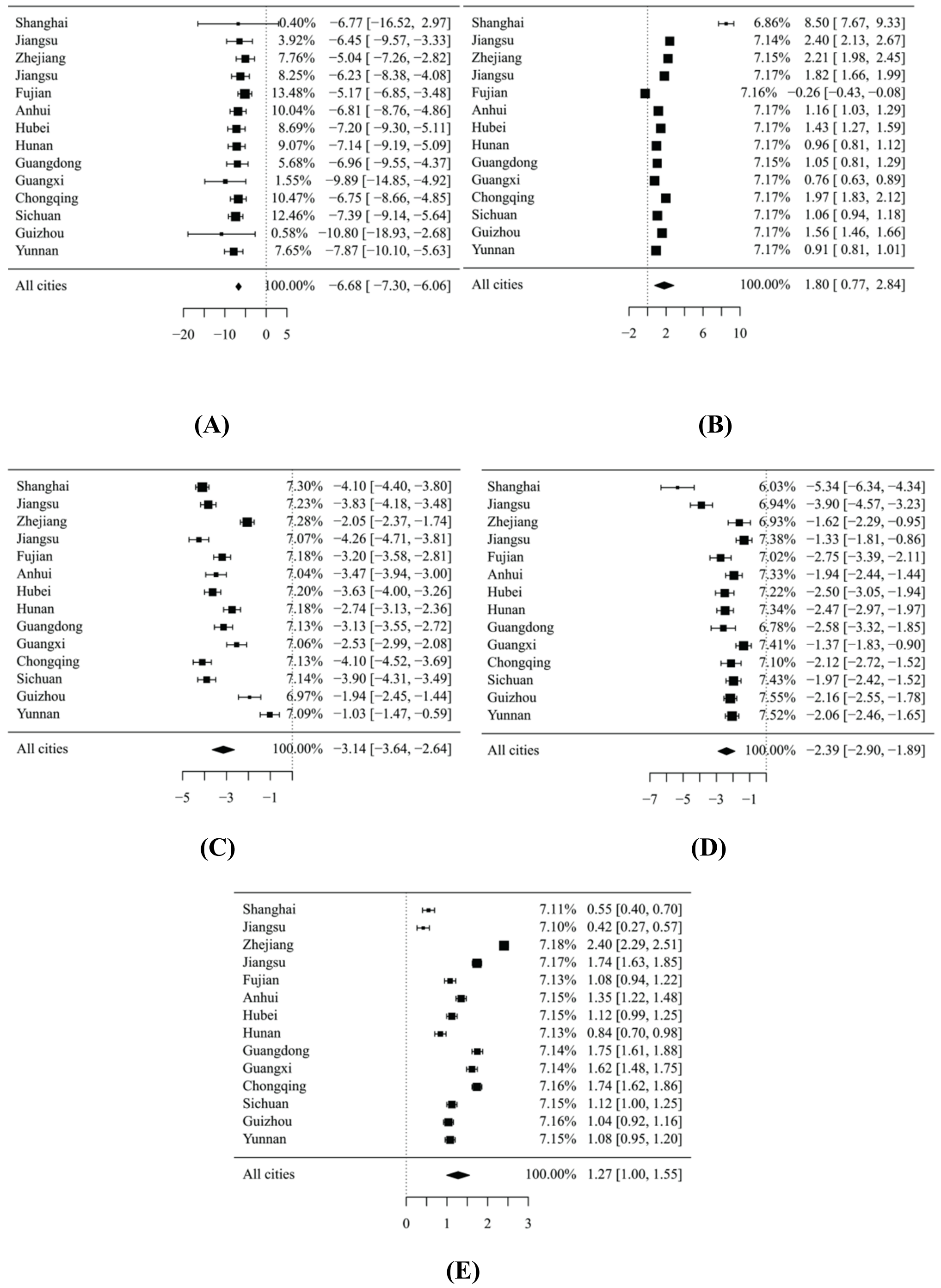

Supplementary Figure S3. Meta-analysis of different types of infectious diarrhea in subtropical monsoon region.(A) Cholera; (B) dysentery; (C) typhoid and paratyphoid; (D) other infectious diarrheas; (E) infectious diarrhea.

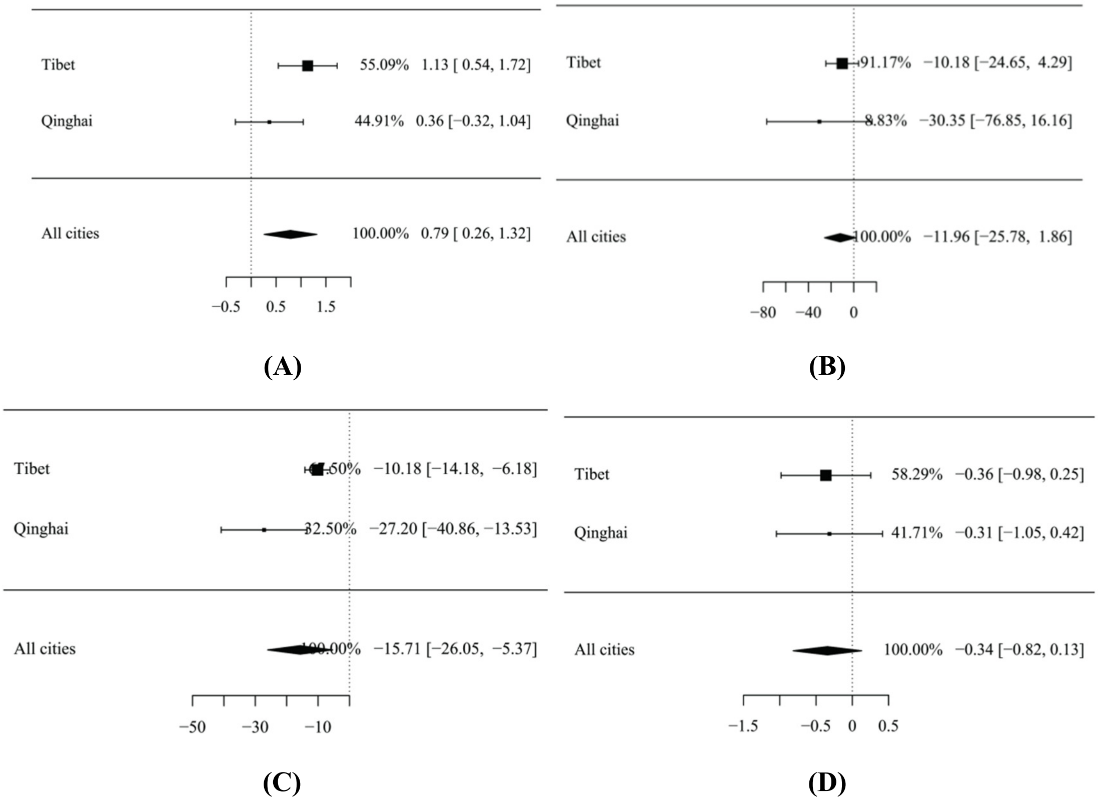

Supplementary Figure S4. Meta-analysis of different types of infectious diarrhea in alpine plateau region. (A) Dysentery; (B) typhoid and paratyphoid; (C) other infectious diarrheas; (D) infectious diarrhea.

Sensitivity analysis

Supplementary Table S12. Parameters from Poisson regression model for the incidence rate of cholera in different climate zones from 2004 to 2018.

	Coefficient	Std. err.	p-value	R^2
**Temperate region**				0.54
Temperature (lag 1)*	0.3924	0.0376	<0.001	
Rainfall (lag 1)*	0.0052	0.0010	<0.001	
sin(2πt/12)	1.3500	0.2926	<0.001	
Month	-0.0276	0.0045	<0.001	
**Subtropical monsoon region**				0.21
sin(2πt/12)*	-2.3027	0.4534	<0.001	
Month	-0.0313	0.0056	<0.001	

*Lag 1/2 represented the lag effects of 1 or 2 months.

Supplementary Table S13. Parameters from Poisson regression model for the incidence rate of dysentery in China and in different climate zones from 2004 to 2018.

	**Coefficient**	**Std. err.**	**p-value**	**R^2**
**Temperate region**				0.90
Temperature	0.0548	0.0012	<0.001	
Relative humidity (lag 2)*	0.0034	0.0010	0.001	
Rainfall	0.0003	0.0001	0.026	
Sunshine duration (lag 2)*	0.0017	0.0003	<0.001	
sin(2πt/12)	-0.3426	0.0180	<0.001	
Month	-0.0124	0.0005	<0.001	
**Subtropical monsoon region**				0.77
Temperature	0.0526	0.0015	<0.001	
Number of doctors (Per 1,000)	-0.0812	0.0194	<0.001	
Population density	-0.0030	0.0002	<0.001	
sin(2πt/12)	-0.1705	0.0150	<0.001	
Month	-0.0086	0.0005	<0.001	

*Lag 1/2 represented the lag effects of 1 or 2 months.

Supplementary Table S14. Parameters from Poisson regression model for the incidence rate of typhoid and paratyphoid in China and in different climate zones from 2004 to 2018.

	**Coefficient**	**Std. err.**	**p-value**	**R^2**
**Temperate region**				0.72
Temperature	0.0247	0.0018	<0.001	
Extreme sunshine duration (lag 2)*	0.1613	0.0481	<0.001	
Proportion of children aged 0-14	-4.1690	1.2920	0.001	
Population density	0.0009	0.0005	0.050	
sin(2πt/12)	-0.2193	0.0270	<0.001	
Month	-0.0093	0.0004	<0.001	
**Subtropical monsoon region**				0.81
Temperature	0.0408	0.0027	<0.001	
Rainfall (lag 1)*	0.0004	0.0002	0.008	
Proportion of children aged 0-14	5.4160	1.0370	<0.001	
sin(2πt/12)	-0.9541	0.0210	<0.001	
Month	-0.0942	0.0004	<0.001	

*Lag 1/2 represented the lag effects of 1 or 2 months.

Supplementary Table S15. Parameters from Poisson regression model for the incidence rate of other infectious diarrheas in China and in different climate zones from 2004 to 2018.

	**Coefficient**	**Std. err.**	**p-value**	**R^2**
**Temperate region**				0.79
Temperature (lag 1)*	0.0487	0.0018	<0.001	
Sunshine duration (lag 2)*	0.0009	0.0002	<0.001	

Population density	-0.0023	0.0001	<0.001	
Number of doctors (Per 1,000)	0.0309	0.0096	0.001	
sin(2πt/12)	0.1445	0.0244	<0.001	
Month	0.0032	0.0003	<0.001	
**Subtropical monsoon region**				0.61
Temperature (lag 2)*	0.0492	0.0055	<0.001	
Rainfall (lag 2)*	-0.0005	0.0001	<0.001	
Proportion of urbanized population	6.8331	0.6891	<0.001	
sin(2πt/12)	0.2448	0.0615	<0.001	
Month	-0.0014	0.0007	0.035	

*Lag 1/2 represented the lag effects of 1 or 2 months.

Supplementary Table S16. Parameters from Poisson regression model for the incidence rate of infectious diarrhea in China and in different climate zones from 2004 to 2018.

	**Coefficient**	**Std. err.**	**p-value**	**R^2**
**Temperate region**				0.83
Temperature (lag 1)*	0.0561	0.0019	<0.001	
Rainfall (lag 1)*	0.0007	0.0002	<0.001	
Sunshine duration (lag 2)*	0.0010	0.0003	0.002	
Population density	-0.0015	0.0002	<0.001	
Number of doctors (Per 1,000)	0.0274	0.0090	0.002	
sin(2πt/12)	0.1684	0.0310	<0.001	
Month	-0.0012	0.0002	<0.001	
**Subtropical monsoon region**				0.57
Temperature (lag 1)*	0.0242	0.0027	<0.001	
Rainfall (lag 2)*	-0.0008	0.0001	<0.001	
sin(2πt/12)	-0.0799	0.0274	0.003	
Month	0.0018	0.0002	<0.001	
